# Increased natural mortality at low abundance can generate an Allee effect in a marine fish

**DOI:** 10.1098/rsos.140075

**Published:** 2014-10-15

**Authors:** Anna Kuparinen, Jeffrey A. Hutchings

**Affiliations:** 1 Department of Environmental Sciences, University of Helsinki, PO Box 65, 00014, Finland; 2 Department of Biology, Dalhousie University, 1355 Oxford St., PO Box 15000, Halifax, Nova Scotia, B3H 4R2, Canada; 3 Centre for Ecological and Evolutionary Synthesis, Department of Biosciences, University of Oslo, Oslo 0316, Norway

**Keywords:** Atlantic cod, density-dependence, fisheries, overfishing, population growth rate, recovery

## Abstract

Negative density-dependent regulation of population dynamics promotes population growth at low abundance and is therefore vital for recovery following depletion. Inversely, any process that reduces the compensatory density-dependence of population growth can negatively affect recovery. Here, we show that increased adult mortality at low abundance can reverse compensatory population dynamics into its opposite—a demographic Allee effect. Northwest Atlantic cod (*Gadus morhua*) stocks collapsed dramatically in the early 1990s and have since shown little sign of recovery. Many experienced dramatic increases in natural mortality, ostensibly attributable in some populations to increased predation by seals. Our findings show that increased natural mortality of a magnitude observed for overfished cod stocks has been more than sufficient to fundamentally alter the dynamics of density-dependent population regulation. The demographic Allee effect generated by these changes can slow down or even impede the recovery of depleted populations even in the absence of fishing.

## 2. Introduction

Negative density-dependent population dynamics promotes population growth at low abundance [1]. A reduction in the strength of this compensatory density-dependence can negatively affect *per capita* population growth, thus inhibiting recovery. Predation, for example, is known to vary with prey abundance, such that a declined prey population can experience elevated rates of natural mortality at low abundances (type-II functional response [2]). It has been postulated that such a shift in predation pressure was experienced by some severely depleted Northwest Atlantic cod (*Gadus morhua*) stocks that have since shown few signs of recovery [3–5], particularly in the Southern Gulf of St. Lawrence, where mortality unrelated to fishing has more than tripled during the past two decades [4,6]. This high rate of natural mortality is capable of driving a cod stock to extinction, even in the absence of fishing [7].

While much of the previous research on mortality changes in cod has focused on quantifying mortality increases and identifying their correlates [4–6] as well as projecting future population development [7], here we approach the topic by considering the potential relevance of an Allee effect. Specifically, we investigate whether an increase in adult mortality at low abundance is a mechanism that can reverse compensatory population dynamics into its opposite, a demographic Allee effect. A demographic Allee effect is characterized by a shift from a negative to a positive correlation between *per capita* population growth rate and a metric of population abundance, as the latter declines [8]. In other words, a population that experiences a demographic Allee effect has lowered growth ability at low abundance. Lack of recovery of many overfished stocks [9] has drawn attention to the question of how common Allee effects might be in marine species and to what extent they can impede the recovery [10]. One means by which Allee effects can be manifest is through interspecific interactions [11]. Whether an Allee effect can be generated by one such interaction—predation—is a fundamental question that might illuminate not only the general mechanisms underlying a population’s or species’ ability to recover from depletion but also causes that could potentially account for the lack of recovery of Northwest Atlantic cod.

## 3. Material and methods

To explore how population dynamics respond to increased predation at low abundance, we used an individual- and process-based modelling approach parametrized for Newfoundland’s northern stock of Atlantic cod (for detailed description of the model and its parametrization, see [12]). The model describes individual life histories through von Bertalanffy (VB) growth curves and known correlations between VB parameters, length at maturity and asymptotic body size. At each time step (year), the model simulates the demographic processes of mortality, growth, maturation and reproduction on an individual basis. Demographic stochasticity is accounted for in each of the processes. The genetic basis of the life histories is modelled through 10 diploid loci and their classical Mendelian inheritance. The model incorporates density-dependence in growth and reproduction. Juvenile production is compensatory such that it increases at low population abundances in accordance with a Beverton–Holt stock-recruitment curve for cod (electronic supplementary material, figure S1) [13].

We partitioned instantaneous rates of adult natural mortality into an overall mortality parameter (*M*=0.12) experienced by all individuals from age 3 years onwards and a parameter representing the survival cost of reproduction (SC=0.1), experienced by all mature individuals. At abundances below 20% of population carrying capacity (*K*), we increased *M* by 0, 50, 75 or 100% to mimic four scenarios of predation-mortality increase. Cod population dynamics were simulated at annual time steps. The population was first exposed to fishing (*F*=0.25; using a logistic selectivity curve, based on body size) until it had declined below 5% of *K*, after which fishing ceased. During the subsequent recovery period, *per capita* population growth rates were recorded for each cohort, as well as the population biomass in the year the cohort was born. Simulations were replicated with versions of the model that did and did not allow for the exploited cod population to evolve in response to changes in mortality. For the former, life histories could evolve during the fishing and recovery periods, whereas life histories remained unchanged in the non-evolving simulations.

## 4. Results

**Figure 1.**
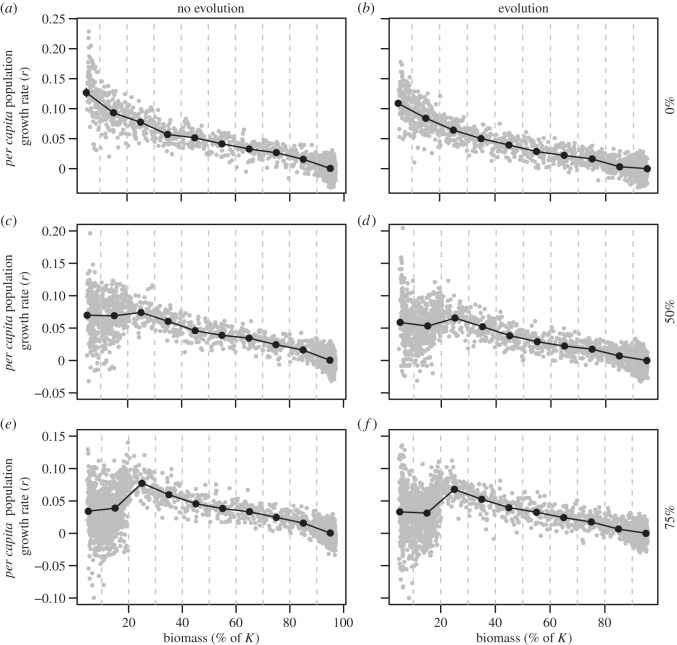
Projected *per capita* population growth rates (*r*) at varying population abundances. Values of *r* estimated for individual cohorts are plotted as grey dots against the population biomass (expressed as % of carrying capacity, *K*) in the year the cohort was born. The average growth rates calculated for abundance ranges 0–10% of *K*,…,90–100% of *K* are shown with black dots, and the black vertical lines encompass 95% CIs (many of these are not readily discernable because they overlap with the dot). Scenarios for the proportional increase in natural mortality at abundances below 20% of *K* are indicated on the right of the panels and scenarios for evolution/no evolution are identified at the top of each set of panels; each panel represents the results of 20 replicated simulation runs. Simulations with 100% mortality increase did not recover (figure 2) and, therefore, a similar *r*-abundance plot could not be drawn for this scenario.

Following the relaxation of fishing, populations began to rebuild towards their equilibrium levels. In the absence of mortality increase at abundances below 20% of *K*, realized population dynamics reflected the underlying compensatory juvenile production, as shown by high *per capita* population growth rates (*r*) at very low abundance (figure 1*a*,*b*). However, as mortality at low abundance increased, compensation weakened and population dynamics shifted to become depensatory such that an Allee effect became evident. This effect is shown by the values of *r* plotted against abundance: when looking at low abundances, a mortality increase of 50% yielded a relatively flat pattern (figure 1*c*,*d*), whereas an increase of 75% caused the values of *r* to drop as the abundance declined (figure 1*e*,*f*). These patterns were similar in both the evolving and non-evolving simulations, although the average values of *r* were often slightly lower in the evolving simulations.

**Figure 2.**
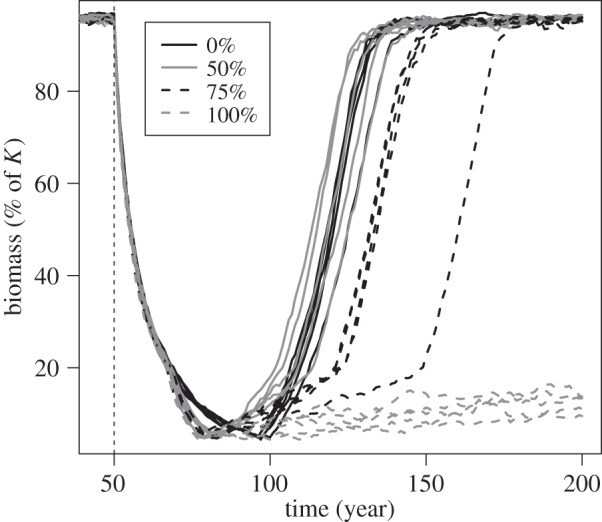
Temporal changes in population biomass during the fishing and recovery periods. Mortality-increase scenarios are indicated by the key and five randomly chosen replicated simulation runs are shown for each scenario. Simulations illustrated here were conducted with the non-evolutionary model version. The dashed vertical line indicates the beginning of fishing, which was continued until the population had declined to 5% of its carrying capacity, *K*.

Despite lower *per capita* growth rates owing to increased mortality, populations experiencing a 50% mortality increase rebuilt their biomass at a pace similar to that of populations that did not experience a change in mortality (figure 2). By contrast, when mortality increased by 75%, biomass rebuilding was severely delayed; when mortality increased by 100%, population levels had attained only 10–15% of *K* after 100 years of rebuilding (figure 2).

## 5. Discussion

Our results suggest that increased mortality at low abundance can generate a demographic Allee effect that slows the recovery of depleted populations. In addition to the general importance of this finding for threatened species, our work has specific relevance to the rebuilding of over-exploited populations, indicating that recovery can be significantly retarded even in the absence of exploitation. Elevated natural mortalities have been documented in four Northwest Atlantic cod stocks following their collapse in the early 1990s [5]. The mortality-increase scenarios explored here are quite conservative when compared with those estimated for the collapsed stocks. In the Southern Gulf of St. Lawrence, *M* is estimated to have more than tripled during and following a period of population reduction [4]. A similar pattern of association between population size and natural mortality is evident for northern cod, which declined by an estimated 99% between 1962 and 1992 [3]. When compared with a natural mortality of less than 0.25 in the 1970s and 1980s, *M* is estimated to have doubled in the 1990s; this increase in natural mortality persisted for more than decade, despite massive reductions in fishing mortality [14].

The findings of this study suggest that the mortality increases experienced by some Northwest Atlantic cod were sufficient to erode compensatory density-dependence in population dynamics to the extent that *per capita* population growth, and thus recovery rate, was severely reduced. This finding is inconsistent with the perception that Allee effects are not commonly present in marine fish and that their recovery ability should generally not be compromised by low abundance [13,15]. While this perception arose because of the challenges in detecting Allee effects (due to lack of statistical power in data and analyses), more recently it has been acknowledged that marine fish populations reduced to very low levels of abundance might indeed experience Allee effects [16]. This study provides an example of the underlying mechanisms through which a demographic Allee effect can arise and how it might impede recovery.

The exact causes for increased mortality in some depleted cod populations remain to some extent unclear and debatable, and the amount of unintentional fishing mortality through bycatches is not always well known. Nonetheless, the weight of evidence suggests that natural mortality has increased and that it can be partially attributed to predation of cod by seals [4,17–19]. In eastern Canada, concurrently with a 97% reduction in cod biomass [3], the abundance of grey seals (*Halichoerus grypus*) increased 50-fold [20] while that of harp seals (*Pagophilus groenlandicus*) has increased fourfold [21]. Despite large reductions in cod abundance, the proportion of cod in seal diets in some areas has remained substantial [17,19,22]. This general pattern suggests that relative cod mortality by seals can increase as cod abundance decreases; that is, their predator–prey dynamics exhibit elements characteristic of a type-II or type-III functional response [2]. Such ‘predator pits’, which might not be uncommon in marine ecosystems [23], can be caused by the behaviour of the prey. Southern Gulf of St. Lawrence cod, for example, undertake an autumnal migration to a comparatively restricted over-wintering region [4] where the high density of their aggregations might be sufficient to render them vulnerable to a consistent number of prey being eaten per predator.

Allee effects are clearly problematic, negatively affecting both the time [8] and uncertainty [24] of recovery. This study highlights the synergistic influence that low abundance coupled with increased mortality can have from a conservation perspective. Changes in mortality resulting from altered interspecific interactions—the mechanism proposed here—might not be uncommon. Indeed there is good evidence to suggest that increases in predator-induced mortality might be associated with low abundance in other marine fish [17]. These observations underscore the necessity of evaluating the consequences of overfishing from an ecosystem perspective: prolonged and heavy fishing mortality combined with increasing predator abundance can lower fish population resilience and adversely affect their viability.

## Supplementary Material

ESM Fig.1. Reproductive success as a function of population abundance.

## Data accessibility

Data supporting this article has been uploaded as part of the Supplementary Material.

## References

[1] Murdoch WW 1994 Population regulation in theory and practice. *Ecology* 75, 271–287. (doi:10.2307/1939533)

[2] Murdoch WW 1972 The functional response of predators. *Biol. Control* 15, 237–240.

[3] Hutchings JA, Rangeley RW 2011 Correlates of recovery for Canadian Atlantic cod. *Can. J. Zool.* 89, 386–400. (doi:10.1139/z11-022)

[4] Swain DP 2011 Life-history evolution and elevated natural mortality in a population of Atlantic cod (Gadus morhua). *Evol. Appl.* 4, 18–29. (doi:10.1111/j.1752-4571.2010.00128.x) 25567950 10.1111/j.1752-4571.2010.00128.x PMC3352523

[5] Bundy A, Heymans JJ, Morissette L, Savenkoff C 2009 Seals, cod and forage fish: a comparative exploration of variations in the theme of stock collapse and ecosystem change in four Northwest Atlantic ecosystems. *Prog. Ocean* 81, 188–206. (doi:10.1016/j.pocean.2009.04.010)

[6] Swain DP, Mohn RK 2012 Forage fish and the factors governing recovery of Atlantic cod (Gadus morhua) on the eastern Scotian Shelf. *Can. J. Fish Aquat. Sci.* 56, 997–1001. (doi:10.1139/f2012-045)

[7] Swain DP, Chouinard G 2008 Predicted extirpation of the dominant demersal fish in a large marine ecosystem: Atlantic cod (Gadus morhua) in the southern Gulf of St. Lawrence. *Can. J. Fish Aquat. Sci.* 65, 2315–2319. (doi:10.1139/F08-175)

[8] Stephens PA, Sutherland WJ, Freckleton RP 1999 What is the Allee effect?. *Oikos* 87, 185–190. (doi:10.2307/3547011)

[9] Pauly D, Christensen V, Dalsgaard J, Froese R, Torres F Jr 1998 Fishing down marine food webs. *Science* 279, 860–863. (doi:10.1126/science.279.5352.860) 9452385 10.1126/science.279.5352.860

[10] Gascoigne J, Lipcius RN 2004 Allee effects in marine systems. *Mar. Ecol. Prog. Ser.* 269, 49–59. (doi:10.3354/meps269049)

[11] De Roos AM, Persson L 2002 Size-dependent life-history traits promote catastrophic collapses of top predators. *Proc. Natl Acad. Sci. USA* 99, 12907–12912. (doi:10.1073/pnas.192174199) 12237404 10.1073/pnas.192174199 PMC130558

[12] Kuparinen A, Hutchings JA 2012 Consequences of fisheries-induced evolution for population productivity and recovery potential. *Proc. R. Soc. B* 279, 2571–2579. (doi:10.1098/rspb.2012.0120) 10.1098/rspb.2012.0120 PMC3350703 22398166

[13] Myers RA, Barrowman NJ, Hutchings JA, Rosenberg AA 1995 Population dynamics of exploited fish stocks at low population levels. *Science* 269, 1106–1108. (doi:10.1126/science.269.5227.1106) 17755535 10.1126/science.269.5227.1106

[14] DFO. 2013 *Stock assessment of northern (2J3KL) cod in 2013 Canadian Science Advisory Secretariat Science Advisory Report 2013/014.* Ottawa, Canada: Department of Fisheries and Oceans Canada.

[15] Liermann M, Hilborn R 1997 Depensation in fish stocks: a hierarchical Bayesian meta-analysis. *Can. J. Fish Aquat. Sci.* 54, 1976–1984. (doi:10.1139/f97-105)

[16] Hutchings JA 2014 Renaissance of a caveat: Allee effects in marine fish. *ICES J. Mar. Sci.* 71, 2152–2157. (doi:10.1093/icesjms/fst179)

[17] Benoît HP, Swain DP, Bowen WD, Breed GA, Hammill MO, Harvey V 2011 Evaluating the potential for grey seal predation to explain elevated natural mortality in three fish species in the southern Gulf of St. Lawrence. *Mar. Ecol. Prog. Ser.* 442, 149–167. (doi:10.3354/meps09454)

[18] DFO. 2010 *Impact of grey seals on fish populations in eastern Canada. Canadian Science Advisory Secretariat Science Advisory Report 2010/071.* Ottawa, Canada: Department of Fisheries and Oceans Canada.

[19] Hammill MO, Stenson GB, Swain DP, Benoît HB 2014 Feeding by grey seals on endangered stocks of Atlantic cod and white hake. *ICES J. Mar. Sci.* 71, 1332–1341. (doi:10.1093/icesjms/fsu123)

[20] DFO. 2014 *Stock assessment of Canadian grey seals (Halichoerus grypus). Canadian Science Advisory Secretariat Science Advisory Report 2014/010.* Ottawa, Canada: Department of Fisheries and Oceans Canada.

[21] Hammill MO, Stenson GB, Doniol-Valcroze T, Mosnier A 2013 *Estimating carrying capacity and population trends of Northwest Atlantic harp seals, 1952–2012. Canadian Science Advisory Secretariat Science Advisory Report 2012/148.* Ottawa, Canada: Department of Fisheries and Oceans anada.

[22] O’Boyle R, Sinclair M 2012 Seal–cod interactions on the Eastern Scotian Shelf: reconsideration of modelling assumptions. *Fish. Res.* 115–116, 1–13. (doi:/10.1016/j.fishres.2011.10.006)

[23] Bakun A 2006 Wasp-waist populations and marine ecosystem dynamics: navigating the ‘predator pit’ topographies. *Prog. Ocean* 68, 271–288. (doi:10.1016/j.pocean.2006.02.004)

[24] Kuparinen A, Keith DM, Hutchings JA 2014 Allee effect and the uncertainty of population recovery. *Conserv. Biol.* 28, 790–798. (doi:10.1111/cobi.12216) 24512300 10.1111/cobi.12216

