# Supplementary material for: Increased natural mortality at low abundance can generate an Allee effect in a marine fish
Source: R Soc Open Sci. 2014 Oct 15;1(2):140075. doi: 10.1098/rsos.140075 (PMC4448890; doi:10.1098/rsos.140075)

**Fig. 1** Reproductive success as a function of population abundance (% of carrying capacity K). Reproductive success is estimated using recruit-per-spawner ratios predicted by the Beverton-Holt stock-recruitment model for Atlantic cod (Myers et al. 1995) and then scaled by the ratio at the abundance 40 % of K.


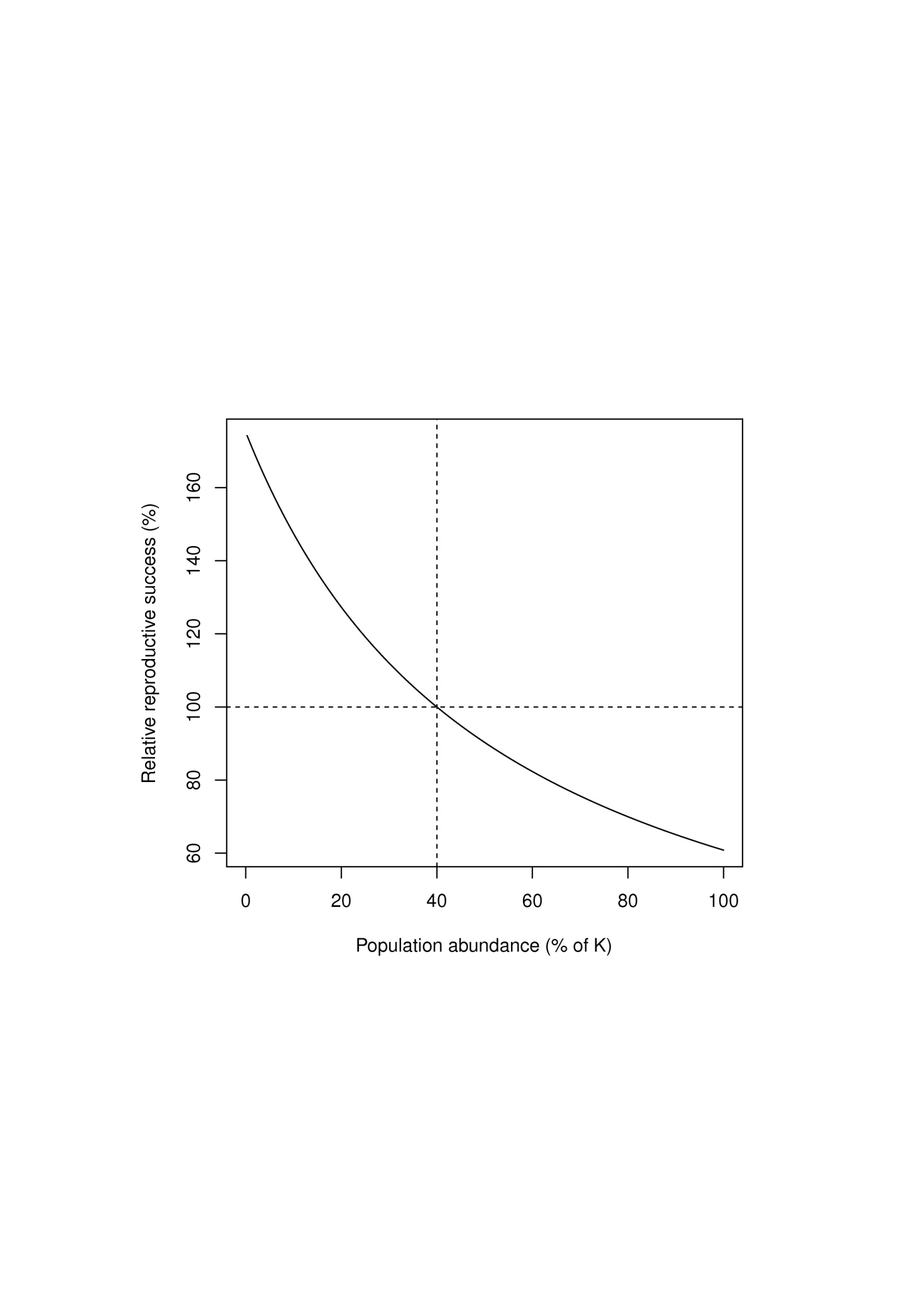

Supplement: ESM Fig.1. Reproductive success as a function of population abundance. [file rsos140075supp1.docx]
